# Can Artificial Intelligence Interpret Pulmonary Function Tests and Predict Prolonged Air Leaks After Lung Resection

**DOI:** 10.3390/cancers18091484

**Published:** 2026-05-05

**Authors:** Omar Zahra, Alexander Pohlman, Ayham Odeh, Mohammad Alhusseini, James Lubawski, Julia M. Coughlin, Wissam Raad, Amit Goyal, Zaid M. Abdelsattar

**Affiliations:** 1 Stritch School of Medicine, Loyola University Chicago, Maywood, IL 60153, USA; ozahra@luc.edu (O.Z.); apohlman@luc.edu (A.P.); ayhamodeh123@gmail.com (A.O.); malhusseini@luc.edu (M.A.); julia.coughlin@lumc.edu (J.M.C.); wraad1@alumni.jh.edu (W.R.); agoyal@lumc.edu (A.G.); 2 Department of Thoracic and Cardiovascular Surgery, Loyola University Medical Center, Maywood, IL 60153, USA; 3 Department of Surgery, University of Illinois Chicago, Chicago, IL 60612, USA; 4 Department of Surgery, Ascension St. Vincent Hospital, Indianapolis, IN 46260, USA; 5 Division of Pulmonology and Critical Care, Department of Medicine, Loyola University Medical Center, Maywood, IL 60153, USA; 6 Department of Veterans Affairs, Edward Hines Jr. VA Hospital, Hines, IL 60141, USA

**Keywords:** lung cancer, artificial intelligence, machine learning, pulmonary function tests, prolonged air leak, lung resection

## Abstract

Background/Objectives: Preoperative pulmonary function tests (PFTs) contain numerous physiologic parameters, yet surgeons typically rely on forced expiratory volume in one second (FEV_1_) and diffusing capacity of the lung for carbon monoxide (DLCO) to assess surgical risk. This study aimed to evaluate whether artificial intelligence (AI) could utilize more PFT data to predict the occurrence of prolonged air leak (PAL) following lung resection. Methods: An optical character recognition (OCR) model was used to extract structured data from PFT reports. These data were combined with clinical and demographic features from our institutional Society of Thoracic Surgeons General Thoracic Surgery Database (STS-GTSD) between 2016 and 2023. A feature selection algorithm was used to select the most predictive features, and a neural network was trained and tested on an internal validation cohort to predict PAL. Model performance was compared to previously published models. Results: There were 410 patients undergoing lung resection who had PFTs successfully digitized by the OCR system. A total of 76 available PFT features were extracted per patient. The final AI model included 10 key input variables, including three PFTs and seven clinical variables. On validation, the model achieved a specificity of 73%, sensitivity of 60%, overall accuracy of 72%, and an area under the curve of 0.74. This performance exceeded most existing PAL prediction models. Conclusions: AI-driven models using structured PFT and clinical data can enhance prediction of prolonged air leak after lung resection and outperform conventional regression-based models. Further research may focus on external validation and integration into clinical workflows.

## Simple Summary

Lung resection is the gold standard of treatment for lung cancer. Prolonged air leaks are the most common complication after lung resection and can significantly prolong hospital stays and increase healthcare costs. Prolonged air leaks are characterized by the continued leakage of air from the lungs for more than five days. There are currently few and largely inaccurate predictors to estimate the risk of prolonged air leaks. Most of these predictive models use only a few clinical measures and breathing tests known as pulmonary function tests. These breathing tests provide many details about lung function, but surgeons often only account for two of these measures. In this study, we use artificial intelligence to interpret pulmonary function tests and improve the prediction of prolonged air leaks after lung cancer resection. Our results reveal a higher accuracy than existing prediction models, indicating that artificial intelligence may help improve the assessment of surgical risks in lung surgery.

## 1. Introduction

Prolonged air leaks (PAL) are a common and clinically significant postoperative complication following pulmonary resection, defined as the persistence of air leakage beyond five days after surgery. According to an analysis of The Society of Thoracic Surgeons General Thoracic Surgery Database, PAL occurs in approximately 10–15% of patients undergoing pulmonary resections [1]. PAL is associated with prolonged hospital stays, increased rates of postoperative infections, elevated healthcare costs, and worsened patient outcomes [2,3,4]. Early identification and risk stratification of patients at elevated risk for PAL are critical for optimizing perioperative management strategies and improving clinical outcomes.

Pulmonary function tests (PFTs) have always been a cornerstone in the preoperative workup for lung resections, providing essential measurements of lung volume, airflow, and gas exchange to evaluate surgical risk [5,6]. Several studies support the critical role of PFTs in assessing operative fitness, although some have reported conflicting findings regarding their predictive accuracy for postoperative complications [7,8,9]. Predictive algorithms have been developed, mostly utilizing logistic regression, to estimate the risk of post-lung resection prolonged air leaks, with multiple models derived from large registries, such as the Society of Thoracic Surgeons General Thoracic Surgery Database (STS-GTSD) or Italian VATS registry, showing variable results [1,10]. Simultaneously, the application of artificial intelligence (AI) and machine learning to preoperative clinical data has emerged as a promising approach to predict disease states and surgical complications [11,12]. This may be particularly relevant when combined with preoperative PFTs. For example, a prior study developed an AI model that demonstrated equivalent or better diagnostic accuracy of an AI’s interpretation of PFTs when compared to pulmonologists alone [13].

Despite these efforts, no PAL prediction models have demonstrated adequate validity or consistent accuracy for clinical application. Surgeons traditionally rely on forced expiratory volume in one second (FEV_1_) and diffusing capacity for carbon monoxide (DLCO) as the principal pulmonary function test parameters to predict postoperative outcomes [14]. However, emerging evidence suggests that other PFT variables may also provide valuable insights into postoperative complication risks [15]. While AI applications in medicine are promising, they remain in early phases of adoption and are not yet established as standard diagnostic tools [11]. Nonetheless, ongoing advancements indicate significant future potential for AI and other clinical metrics in enhancing prediction and decision-making in thoracic surgery [10,16,17,18].

In this context, we aim to use available preoperative PFT parameters with artificial intelligence to predict the risk of prolonged air leaks following pulmonary resection. Specifically, we hypothesize that (1) AI can accurately interpret PFTs from the electronic health record; and (2) AI can outperform previously published risk prediction models. Our findings contribute further evidence to the potential role of AI in enhancing preoperative risk stratification, refining existing predictive models, and ultimately reducing surgical morbidity and mortality.

## 2. Materials and Methods

### 2.1. Data Source and Patient Population

De-identified patient data were obtained from our institutional Society of Thoracic Surgeons General Thoracic Surgery Database (STS-GTSD) at Loyola University Medical Center [19]. A consecutive cohort of adult patients (≥18 years old) who underwent lung resection, including wedge resections, segmentectomy, and lobectomy for known or suspected malignancy between 2016 and 2023 were included, representing the most recently complete annual dataset available at the time of study initiation. All patients were required to have available preoperative pulmonary function test (PFT) data and documented postoperative outcomes. Patients were excluded if they underwent non-pulmonary thoracic procedures only (e.g., esophagectomy), pneumonectomy or sleeve resections, had missing or incomplete PFT data, underwent emergency surgery, were undergoing reoperation without new PFTs, died within five days postoperatively, or lacked clearly documented resection procedures.

### 2.2. Independent Variables and Outcome Measures

Preoperative data was collected, including patient demographics and clinical characteristics, such as age, sex, race, ethnicity, smoking history (reported as number of pack-years), PFT results, and comorbid conditions (e.g., COPD, diabetes, and hypertension). Operative details included the type of resection (wedge, segmentectomy, or lobectomy) and surgical approach (robotic, video-assisted thoracoscopic [VATS], or open). Patients’ zip codes and insurance information were also recorded as proxies for socioeconomic status to account for the social determinants of health. The primary outcome was the occurrence of a prolonged air leak following lung resection, defined as an air leak lasting >5 days.

### 2.3. Main Exposure, Image Extraction and Preprocessing

An Optical Character Recognition (OCR) and Python-based algorithm were used to extract structured data from PFT reports, ensuring accurate digitization and integration into the analysis. The extracted data underwent preprocessing steps, including missing value imputation, normalization, and feature scaling to ensure model convergence and performance. Not all patients had all PFT elements measured and there were some differences in reporting of PFT values that precluded combining certain variables across all patients. Thus, individual elements absent in more than 20% of patients were removed, reducing the total feature set from 233 initially extracted features to 76 complete PFT variables; the same was completed for clinical variables, which led to a total of 63 clinical variables (Table S1). Since only variables with fewer than 20% of patients having missing data were included in the analysis and the vast majority of continuous variables were normally distributed (including those used in the final model), those with missing data were imputed using the mean, and all features were subsequently normalized to a standard distribution. Given the low percentage of patients with missing data, this method aimed to maximize the number of included patients, while minimizing bias. Any patients with unknown categorical clinical or demographic variables were logged as other or unknown and included in the analysis.

### 2.4. Artificial Intelligence Model Derivation

To optimize model performance and minimize overfitting, a forward stepwise feature selection algorithm (FSA) was applied to the final pool of 76 PFT variables and 63 clinical variables. FSA is an iterative method used to evaluate how the addition of one feature at a time affects model accuracy via area under the curve (AUC). At each step, features were added based on performance gains with 4-fold cross-validation used to monitor for overfitting. The optimal feature subset was determined by identifying the point at which the Sequential Feature Selection performance curve plateaued (Figure S1). Beyond 10 features, additional variables yielded marginal improvements in model performance. Consequently, these top 10 variables were selected for the final deep neural network (DNN) architecture, ensuring a model that captures the primary predictive signals while minimizing the risk of over-complex co-adaptation. Clinical relevance of variables was also confirmed by clinician investigators. This hybrid approach combining algorithmic selection with domain expertise ensured inclusion of features that were both statistically optimal and clinically meaningful, improving both interpretability and generalizability. FSA plays a critical role in high-dimensional biomedical modeling, enabling models to focus on the most informative variables, while allowing for complex interactions between variables that may not occur on a linear scale [20,21]. Meanwhile, other feature selection techniques, such as Least Absolute Shrinkage and Selection Operator (LASSO) regression alone may fail to identify nonlinear interactions, as is often seen in clinical scenarios [22]. The final feature set used in the model, including 3 PFT-based variables and 7 clinical/demographic inputs, is detailed in the results section. Statistical tests, including Student’s *t*-test, f-test, and mutual information analysis, confirmed variables that were significantly associated with the primary outcome of prolonged air leak.

### 2.5. Neural Network Model Creation

**Figure 1:**
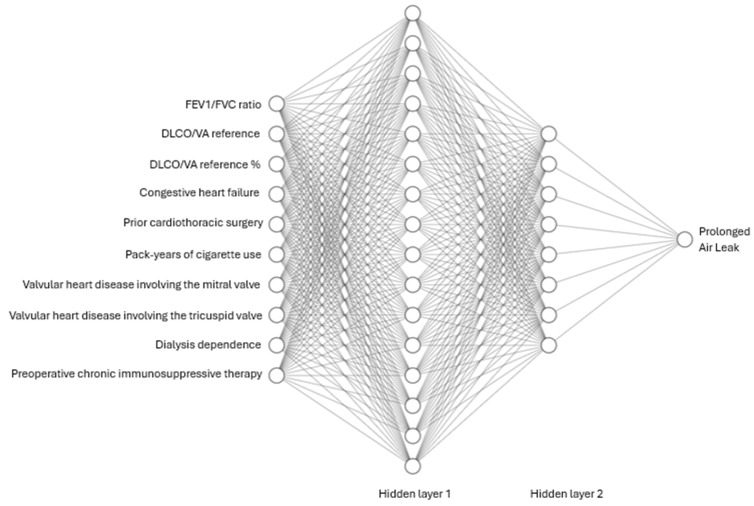
Flow diagram demonstrating the neural network used, including an input layer of 10 nodes, two hidden layers with 16 and 8 nodes, respectively, and an output layer with a single node. Each node is inter-connected with all nodes in the previous and following layers, forming a dense network structure.

A neural network was created using the input variables selected via the FSA above. The architecture of the neural network can be seen in Figure 1; it includes an input layer of 10 nodes, two hidden layers comprising 16 and 8 nodes, followed by an output layer with a single node for binary classification (PAL versus no PAL). To mitigate potential instabilities inherent in forward stepwise selection, we implemented a multi-faceted regularization framework. Within the initial hidden layer of the DNN, an L1 (LASSO) penalty was applied to promote weight sparsity and feature refinement, complemented by a Dropout layer to prevent neuronal co-adaptation and overfitting. We utilized the Adam optimizer to ensure stable and efficient convergence during the weight update process. By integrating sequential feature selection with these internal regularization constraints, our methodology offered a robust, nonlinear alternative to traditional linear LASSO models, specifically engineered to leverage the high-dimensional learning capabilities of a DNN architecture.

Model training and validation was performed using a stratified 4-fold cross-validation approach on the total dataset. For each fold, the data was split into a training cohort (~308 samples) and an independent test cohort (~102 samples) to ensure internal validation. Within each training fold, a further internal split was utilized: 80% of the samples (*n* = 246) were used for weight updates, while the remaining 20% (*n* = 62) served as a hold-out validation set. This validation set was used to monitor performance at the end of each epoch; specifically, an early stopping criterion was applied to halt training once the validation loss ceased to improve, thereby preventing overfitting and ensuring the model retained optimal generalization capabilities. Results were aggregated to create an overall average performance with confidence intervals. The model was trained for a maximum of 200 epochs using a batch size of 16. Model weights were initialized at random prior to training to avoid introducing bias. Backpropagation was used to iteratively adjust weights based on prediction error, allowing the network to optimize its internal parameters [23]. For instance, if the model incorrectly predicted a probability of 0.5 for an event that truly occurred (true value of 1), weight adjustments were applied in a direction that would increase the likelihood toward the correct outcome in subsequent iterations.

### 2.6. Statistical Analysis and Validation

Initial patient demographics were compared between those that did and did not develop prolonged air leaks. Student’s *t*-tests were used for normally distributed continuous variables as determined by a skewness range between −1 and +1; of note, smoking history and all PFTs used in the neural network model were normally distributed. All other input variables were categorical; Chi-square tests were used to compare categorical variables.

The trained artificial intelligence model was evaluated using the internal validation cohort to predict the occurrence of prolonged air leaks. Predictions were determined based on a threshold probability of 0.5 with values ≥0.5 classified as positive (PAL predicted) and values <0.5 classified as negative (PAL not predicted). Model predictions were compared to actual clinical outcomes to determine true positive (TP), true negative (TN), false positive (FP), and false negative (FN) rates. Model performance was further assessed by generating a receiver operating characteristic (ROC) curve and calculating the area under the curve (AUC) to quantify the model’s discriminative ability [23].

## 3. Results

A total of 410 patients underwent lung resection during the study period with pulmonary function tests (PFTs) that were successfully extracted and digitized using the optical character recognition (OCR) model. Of the 410 patients included in the final analysis, 58.1% were female, 79.0% were white, and 75.1% were current or former smokers. Overall, the mean age was 67.1 ± 10.0 years. All resections were performed for known or suspected malignancy, of which, final pathology revealed malignancy on 366 (89.3%) and benign lesions on 44 (10.7%) patients. Several baseline comorbidities were present in this population, including hypertension (68.8%), diabetes (23.7%), and chronic obstructive pulmonary disease (COPD, 11.0%). Less common but clinically significant conditions and medications included immunosuppressive therapy (5.1%), congestive heart failure (4.2%), valvular heart disease (1.5%), and dialysis dependence (1.0%).

Among these patients, 12.2% (*n* = 50) experienced a prolonged air leak (PAL), while 87.8% (*n* = 360) did not. PFT analysis revealed a mean forced expiratory volume in 1 s (FEV_1_) of 2.1 ± 0.66 L, and a mean forced vital capacity (FVC) of 3.08 ± 0.85 L, with an average FEV_1_/FVC percent predicted of 69.7 ± 10.7. The mean peak expiratory flow (PEF) was 5.8 ± 1.8 L/s, and the mean diffusing capacity for carbon monoxide (DLCO) was 16.1 ± 5.1 mL/min/mmHg, with an average of 76% predicted. Total lung capacity (TLC) averaged 5.5 ± 1.4 L, while the residual volume to TLC ratio (RV/TLC) was 40.8 ± 10.1.

Demographics and clinical characteristics for patients who had a PAL and those who did not are summarized in Table 1. PAL patients were more likely to be older (70.2 years vs. 66.7 years, *p* = 0.019), have higher tobacco exposure (40.9 pack-years vs. 26.8, *p* = 0.001), higher rates of coronary artery disease (32% vs. 17.8%, *p* = 0.017), peripheral vascular disease (10% vs. 3.3%, *p* = 0.027), chronic obstructive pulmonary disease (28% vs. 8.6%, *p* < 0.001), and have a history of prior cardiothoracic surgery (26.0% vs. 10.0%, *p* = 0.001). Hypertension, diabetes, and other systemic diseases were similar between both groups. Surgical aspects were also associated with significant differences. For example, lobectomies were more common in the PAL group compared to those without PAL (68% vs. 45.4%); meanwhile wedge resections were less common in the PAL group (20% vs. 43.2%, *p* = 0.004). Together, these findings demonstrate the complex interplay between baseline risk factors and operative characteristics.

During FSA, 10 key variables were identified as predictive inputs for the model. Among these, three originated from PFTs and seven were identified from clinical data. PFT variables included FEV1/FVC ratio, predicted Diffusing Capacity of the Lungs for Carbon Monoxide adjusted for Alveolar Volume (DLCO/VA reference), and the percent of predicted DLCO/VA ratio. Clinical variables included congestive heart failure, prior cardiothoracic surgery, pack-years of cigarette use, valvular heart disease involving the mitral valve, valvular heart disease involving the tricuspid valve, dialysis dependence, and preoperative chronic immunosuppressive therapy. This selection aimed to balance physiologic predictors with patient-level risk factors to optimize the neural network’s predictive accuracy [24,25].

**Figure 2:**
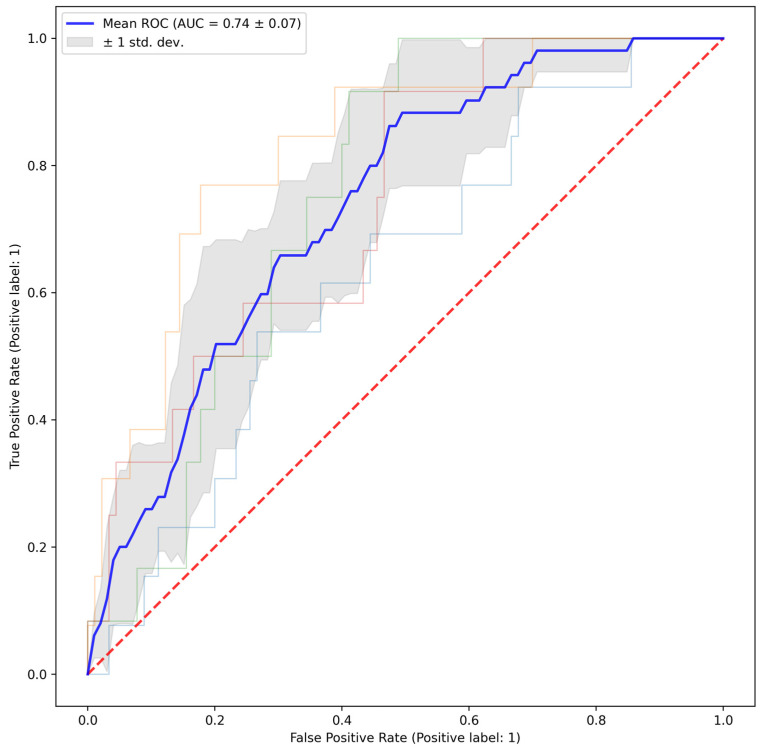
The Receiver Operating Characteristic (ROC) curve displays the trade-off between the true positive rate (sensitivity) and the false positive rate (1-specificity) for the classifier. The curve represents the classifier’s performance across various threshold settings, with an Area Under the Curve (AUC) of 0.74, indicating a relatively strong discriminative ability.

The model consisted of 345 total trainable parameters, which represent the coefficients/weights applied to every individual connection between all nodes of the neural network. Another way of understanding this would be to say that in a simple linear regression there are only two trainable parameters, but in a neural network, such as this, there are several hundred that allow for more nuanced calculations and accurate risk prediction. Reliability of the model before and after calibration can be seen in Figure S2. The pre-calibration Brier score was 0.187 ± 0.016, and the post-calibration Brier score was 0.102 ± 0.005, indicating a highly reliable model, particularly for lower probability ranges where the majority of observations reside; the plateau seen at higher probability thresholds is attributed to the inherent class imbalance, but given the low overall Brier score, the model maintains high predictive utility across the test set. Overall, the model had a sensitivity of 60.1% (95% CI: 46.0–74.2%), a specificity of 73.3% (95% CI: 70.1–76.5%), and an overall accuracy of 71.7% (95% CI: 70.6–72.8%) in the validation set. The Receiver Operating Characteristic (ROC) curve, shown in Figure 2, depicts the model’s ability to distinguish between patients with and without prolonged air leak [23]. The area under the curve (AUC) was 0.74, indicating strong discriminative capability.

In the final regularized model, SHAP values were calculated to determine the individual effects of each input variable. Six of the ten values had zero or near-zero effects, indicating that the L1 penalty effectively neutralized them. The four variables that contributed most to the model’s predictions can be seen in Figure S3. These included the three PFT variables and pack-years of smoking history.

When comparing this model to other previously published models for predicting prolonged air leak, as shown in Table 2, it was noted that the discriminative ability (AUC) of the proposed neural network was higher than most previously published models.

## 4. Discussion

In this study, we utilized preoperative pulmonary function test results combined with clinical characteristics for the development and validation of a neural network-based model to predict prolonged air leak (PAL) after lung resection. In our investigation, we found: (1) AI could effectively utilize PFT data obtained via scanned documents using an optical character recognition program; (2) the AUC of our model was 0.74 indicating that it had a better discriminative ability compared to most prior logistic regression models; and (3) the PFT variables used in creating our model were not the same as many of those used in routine clinical practice when determining risk stratification. Therefore, AI may provide significant benefits toward improving preoperative risk stratification for prolonged air leaks after lung resection.

Regression-based models created from sizable clinical databases, such as the Society of Thoracic Surgeons-General Thoracic Surgery Database and European Society of Thoracic Surgeons database, have historically been used to predict PAL risk, but they have only had moderate predictive accuracy [1,26]. In more recent research, machine learning algorithms have been used to predict postoperative complications with greater accuracy [10,12,28]. This accuracy can depend heavily on the selected prediction variables and models. For example, two prior studies utilizing large databases (STS-GTSD and the European STS database) both encompassed broad samples ranging from 5000–50,000 patients; they both relied upon logistic regression models using commonly known predictors of air leaks, such as low body mass index, low FEV1 (<70%), male sex, right upper lobe resection, and larger resection extents, such as lobectomy [1,26]. However, despite these models being developed on large samples, they had relatively low accuracy and discriminative capability. It is possible that this could be due to greater heterogeneity in those studies as they encompassed multiple institutions, but some also limited the samples to certain resections, such as the European STS study that limited the sample to only lobectomies. Meanwhile, another study using machine learning methods on a sample of over 6000 patients used a combination of these features including one feature identified in our study: DLCO/VA ratio [10]. However, after internal validation, that model only reached an AUC of 0.63, which the authors determined was too low for clinical practice. Other machine learning models have looked at broader pulmonary complications, while comparing several different machine learning methods, with the highest achieving an AUC of 0.82 [28]. Thus, it is clear that both appropriate feature selection and neural network modeling are vital to creating the most accurate peri-operative risk prediction tools.

In this study, the AUC of 0.74 for our model suggests favorable predictive capabilities when combined with PFT data. Although the model sensitivity of 60% may sound low, similar logistic regression models trained on thousands of patients had lower sensitivities of 30–37% [1,26], so it is likely that a larger training set in the future would allow for improved accuracy of the model and greater confidence in clinical integration. Additionally, the specificity of 73%, which translates into a negative predictive value of 93% indicates that the model could be utilized as a tool for determining who is unlikely to develop a PAL rather than determining who will. Regardless, further training and validation in larger study populations will be helpful in improving its accuracy before utilization as a screening tool.

Outside of prolonged hospital stays, there has been some discussion on the predictive value of pulmonary function tests in relation to postoperative complications. PFTs have been emphasized in preoperative evaluation according to current guidelines [14,29], but previous studies have reported conflicting results on their predictive value for other postoperative complications [7,8,9]. It is feasible that similar models could be used to predict other postoperative complications as well. It is likely that these would require new analyses and different input variables specific to those complications, but this study serves as proof of concept towards that ultimate goal. Artificial intelligence models are often able to detect patterns and trends that cannot be seen by simple statistical tests or visual interpretation by a surgeon if PFTs are used individually [30,31]. The multiple physiological and clinical parameters used in our model allow the artificial intelligence model to form deeper connections and patterns that allow for more accurate risk prediction.

Of note, we found that the variables identified by our feature selection algorithm were different from those most studies and surgeons emphasize in practice. For instance, in clinical practice, intuitive factors such as percent predicted FEV1 and DLCO are the primary measures surgeons use to risk-stratify patients and determine suitability for surgery. In our model, PFT variables including DLCO/VA reference, FEV1/FVC ratio, and percent predicted DLCO/VA, in addition to pack-year smoking history, were found to contribute most to the accuracy of our artificial intelligence model. While it may seem clinically uninterpretable to use the reference DLCO/VA, this feature kept being selected in all iterations of the model. One explanation to this is that this numeric value is a condensed derived value that represents the patients’ age, sex, race and height. Which also then explains the absence of these variables from the rest of the model. To the human eye, this value alone is not readily interpretable, but to an artificial intelligence model it is very useful in prediction generation. Thus, the model utilizes integrated physiologic predictors rather than isolated factors. Similarly, FEV1/FVC and percent predicted DLCO/VA also incorporate multiple pulmonary function measures into composite variables. These ratios may be difficult for a surgeon to easily interpret at face value, but AI allows for more complex pattern formation. As we saw in this study, these measures may be particularly important in the setting of prolonged air leaks where small impairments in alveolar–capillary unit function and lung parenchymal integrity, as quantified by these composite measures, may impact the healing process and the degree of air leak. The prioritization of DLCO/VA suggests that the biological health of the lung parenchyma, specifically its perfusion and gas exchange efficiency, is more predictive of PAL than simple mechanical airflow. Unlike FEV1, DLCO/VA may serve as a replacement for the lung’s underlying tissue quality and its capacity for an effective injury response and healing, both of which are essential to sealing alveolar defects post-resection [32].

Additionally, our FSA identified seven clinical variables that were considered to be important outside of PFT variables, so we decided not to restrict the model to only PFT features. Clinical variables selected by our FSA were ones that may not be typically used, and some may not be mutually exclusive. For example, there may be correlations between congestive heart failure, valvular disease, and prior cardiothoracic surgery. Although these variables may be correlated, each variable may capture a slightly different impact on patient outcomes or nuanced interactions in a patient’s history. A major benefit of neural networks is that they allow for interaction between variables, whereas typical logistic regressions rely on mutually exclusive data points that can create inflation effects if multicollinearity exists. However, it is also important to note that after fitting our model and calculating SHAP values on all variables, it was ultimately revealed that, of all clinical variables, pack-years of smoking history was the only one that seemed to be contributing significantly to model outputs. Thus, despite our FSA selecting several other clinical variables, it is possible that future models could function well without them.

Previous work in machine learning has similarly demonstrated that such models can detect patterns and correctly predict postoperative complications [33]. Within thoracic surgery, prior machine learning models have been developed to predict postoperative cardiac and pulmonary complications using a variety of inputs, including patient demographics, clinical history, laboratory studies (i.e., blood counts), preoperative DLCO, intra-operative vital signs, and procedure-associated records [28,34,35]. Our study expands on this by now using entire PFT results to identify the most important and predictive factors outside of those most commonly used (i.e., DLCO and FEV1), allowing for a more accurate prediction model from a wider array of data.

It is important to note that this study shows the successful development of an AI prediction tool, but it has not yet been implemented into clinical practice. The successful integration of any AI into clinical practice will depend on the level of trust, evidence, and ease of integration into existing workflows. The clinical value of AI tools lies in their potential to provide additional information that is not only predictive but also understandable. In this case, we developed an established and internally validated tool for predicting prolonged air leaks. However, given that this model was trained on data from a single institution, this limits its generalizability to other hospitals and patient populations. Moreover, the study sample is from a single US institution that serves an urban/dense suburban demographic which could limit its generalizability to other demographics.

This study has a few limitations that should be taken into consideration. For one, artificial intelligence presents a ‘black box’ where users cannot see how each variable in the model contributes to and interacts with other variables to arrive at a specified outcome. However, the SHAP values presented based on the final model revealed that the three PFT variables and smoking history provided the most predictive information. Second, our sample size may limit the inclusion of other variables. As discussed, we saw a plateau in model performance with the addition of more than 10 variables. It is possible that the predictive power of our model could increase with the addition of more variables. However, addition of more variables in our model could lead to overfitting given our sample size, so a larger sample may allow for improved accuracy and the potential for inclusion of more PFT and clinical variables. Additionally, mean imputation was used for any missing continuous values, which could reduce variance and potentially introduce bias. Third, as discussed previously, the single-institution nature of the study limits its generalizability, so future external validation will be required. Finally, we could not incorporate complex radiographic features and intraoperative factors, which may also play a role in the development of prolonged air leaks. Future incorporation of these features may allow for improved accuracy when accounting for anatomic or operative complexity of a specific patient [30]. Notwithstanding these limitations, our results remain clinically meaningful as our artificial intelligence model outperformed most available models seen in the literature. Future expansion and validation will be vital in making this platform widely accessible to all practicing thoracic surgeons.

## 5. Conclusions

In summary, this study demonstrates the potential of artificial intelligence trained on structured preoperative pulmonary function test data and clinical variables to accurately predict the risk of prolonged air leak after lung resection. By achieving higher performance metrics than traditional regression-based models, this approach highlights the value of artificial intelligence in enhancing perioperative risk assessment. Integrating AI tools with structured physiologic data offers surgeons meaningful insight for operative planning and personalized patient counseling and risk stratification. Future research should focus on external validation, refinement of predictive inputs, and clinical implementation of AI-guided decision tools across larger, multi-institutional datasets.

## Figures and Tables

**Table 1:** Demographic and clinical comparison between patients who developed prolonged air leak (PAL) and those who did not following lung resection.

	No PAL (*n* = 360)	PAL (*n* = 50)	*p*-Value
**Sex**, Female	212 (58.9%)	26 (52.0%)	0.355
**Age**, mean ± SD	66.7 ± 10.2	70.2 ± 8.1	0.019
**Race**			0.376
White	284 (78.9%)	40 (80%)	
Black	40 (11.1%)	8 (16%)	
Asian	7 (1.9%)	1 (2%)	
Other	29 (8.1%)	1 (2%)	
**Ethnicity**, Hispanic	28 (7.8%)	1 (2%)	0.135
**Primary Payor**			0.721
Private Insurance	108 (30%)	13 (26%)	
Medicare	224 (62.2%)	34 (68%)	
Medicaid/Other Government	28 (7.8%)	3 (6%)	
**Comorbidities**			
Hypertension	245 (68.1%)	37 (74%)	0.395
Congestive Heart Failure	15 (4.2%)	2 (4%)	0.956
Coronary Artery Disease	64 (17.8%)	16 (32%)	0.017
Peripheral Vascular Disease	12 (3.3%)	5 (10%)	0.027
Prior Cardiothoracic Surgery	36 (10.0%)	13 (26%)	0.001
Pulmonary Hypertension	7 (1.9%)	1 (2%)	0.979
Diabetes	87 (24.2%)	10 (20%)	0.516
End-Stage Renal Disease	4 (1.1%)	0 (0%)	0.454
COPD	31 (8.6%)	14 (28%)	<0.001
Interstitial Fibrosis	21 (5.8%)	1 (2%)	0.260
**Cigarette Use**, Mean Pack-years (SD)	26.8 (27.4)	40.9 (28.4)	0.001
**Immunosuppressants preop**	20 (5.6%)	1 (2%)	0.285
**Steroids Preop**	9 (2.5%)	2 (4%)	0.538
**Neoadjuvant therapy**	60 (16.7%)	7 (14%)	0.633
**Pathologic Tumor Size (cm)**			0.265
≤2	193 (53.6%)	28 (56%)	
2.1–3	78 (21.7%)	8 (16%)	
3.1–5	35 (9.7%)	7 (14%)	
5.1–7	6 (1.7%)	3 (6%)	
>7	7 (1.9%)	1 (2%)	
Benign Pathology	41 (11.4%)	3 (6%)	
**Extent of Lung Resection**			0.014
Wedge	160 (43.2%)	10 (20.0%)	
Segmentectomy	32 (8.6%)	6 (12.0%)	
Lobectomy	168 (45.4%)	34 (68.0%)	
**PFT Variables**, mean ± SD			
FEV_1_ Percent Predicted (%)	81.3 ± 19.6	76.8 ± 19.5	0.126
FVC (Liters)	3.1 ± 0.8	3.2 ± 0.9	0.173
FEV_1_/FVC Percent Predicted (%)	70.8 ± 9.9	61.4 ± 12.6	<0.001
DLCO Percent Predicted (%)	76.5 ± 21.4	73.5 ± 16.0	0.349
DLCO/VA Reference	5.0 ± 2.8	4.4 ± 0.6	0.161
DLCO/VA Percent Predicted (%)	83.6 ± 23.6	71.6 ± 17.5	0.001
TLC (Liters)	5.4 ± 1.4	6.0 ± 1.4	0.003
RV/TLC Ratio (%)	40.5 ± 10.2	44.1 ± 8.9	0.013

PAL—prolonged air leak, SD—standard deviation, COPD—chronic obstructive pulmonary disease, PFT—pulmonary function test, FEV_1_—forced expiratory volume in one second, FVC—forced vital capacity, DLCO—diffusing capacity of the lungs for carbon monoxide, TLC—total lung capacity, RV—residual volume.

**Table 2:** Comparative Model Performance for Prediction of Prolonged Air Leaks after lung resection. This table compares the performance of previously published models for predicting prolonged air leaks after lung resection to the present study.

Study	Model Type	Sample Size	AUC	Accuracy	Sensitivity	Specificity	Data Source
**This Study**	**Neural** **Network**	**410**	**0.74**	**72%**	**60%**	**73%**	**Single** **Institution**
Seder et al., 2019 [1]	Logistic Regression	52,198	0.64	79%	30%	85%	STS-GTSD registry
Pompili et al., 2017 [26]	Logistic Regression	5069	0.67	71%	37%	75%	European STS Database
Divisi et al., 2023 [10]	Machine Learning	6236	0.63	NR	NR	NR	Italian VATS Registry
Kim et al., 2017 [27]	Logistic Regression	1060	0.80	65%	60%	67%	Single Institution

AUC—area under curve, STS—society of thoracic surgeons, GTSD—general thoracic surgery database, VATS—video-assisted thoracoscopic surgery, NR—not reported.

## Data Availability

The data presented in this study are available on request from the corresponding author. The data are not publicly available due to institutional restrictions.

## Supplementary Materials

The following supporting information can be downloaded at: https://www.mdpi.com/article/10.3390/cancers18091484/s1, Figure S1. Sequential Feature Selector (SFS) curve for determination of optimal number of features to include in model; Figure S2. Artificial intelligence model reliability curves (A) Before calibration (B) After calibration; Figure S3. SHAP (Shapley Additive exPlanations) values for primary contributors to neural network model; Table S1. List of 76 common pulmonary function test (PFT) variables and 63 clinical variables that were available for ≥80% of patients, and thus, were included in the feature selection algorithm.
