# Supplementary material for: Can Artificial Intelligence Interpret Pulmonary Function Tests and Predict Prolonged Air Leaks After Lung Resection
Source: Cancers (Basel). 2026 May 5;18(9):1484. doi: 10.3390/cancers18091484 (PMC13163025; doi:10.3390/cancers18091484)

Table S1. List of 76 common pulmonary function test (PFT) variables and 63 clinical variables that were available for ≥80% of patients, and thus, were included in the feature selection algorithm. Variables used in the final model are in ***bold**.

PFT Variables (76 Total Features)		Clinical Variables (63 Total Features)	
FVC_Pre	DL/VA_adj_Pre	Age	Valvular heart disease location aortic valve
FEV1_Pre	VA_Pre	Zip code	***Valvular heart disease location mitral valve**
***FEV1/FVC_Pre**	IVC_Pre	Insurance	Valvular heart disease location pulmonic valve
FEF25-75_Pre	BHT_Pre	Sex	***Valvular heart disease location tricuspid valve**
PEF_Pre	DLCO_Ref	Caucasian	Major vascular disease
FET100_Pre	DL_Adj_Ref	African	Permanent neurologic impairment
FIVC_Pre	***DLCO/VA_Ref**	Asian	Neurologic symptoms present
FIF50_Pre	VA_Ref	Native American	Myasthenia gravis
PIF_Pre	DLCO_Pre %Ref	Hawaiian	Liver dysfunction
FVC_Ref	DL_Adj_Pre %Ref	Hispanic	Coexisting cancer
FEV1_Ref	***DLCO/VA_Pre%Ref**	Height (cm)	Preoperative chemotherapy or immunotherapy
FEV1/FVC_Ref	VA_Pre %Ref	Weight (kg)	***Chronic immunosuppressive therapy**
FEF25-75_Ref	FVC_LLN	Weight loss in past three months	Chronic anticoagulation therapy
PEF_Ref	FEV1_LLN	Hypertension	On home O2
FVC_Pre %Ref	FEV1/FVC_LLN	Steroids	Alcohol abuse
FEV1_Pre %Ref	FEF25-75_LLN	***Congestive heart failure**	Dementia or neurocognitive dysfunction
FEF25-75_Pre %Ref	FVC_ULN	Coronary artery disease	Major psychiatric disorder
PEF_Pre %Ref	FEV1_ULN	Peripheral vascular disease	ECOG Score
TLC_Pre	FEV1/FVC_ULN	***Prior cardiothoracic surgery**	***Pack-years of cigarette use**
VC_Pre	FEF25-75_ULN	Preoperative chemo	
IC_Pre	TLC_LLN	Preoperative thoracic radiation therapy	
FRCPL_Pre	VC_LLN	Cerebrovascular history	
ERV_Pre	FRCPL_LLN	Pulmonary hypertension	
RV_Pre	RV_LLN	Diabetes	
RV/TLC_Pre	RV/TLC_LLN	Diabetes therapy	
Vtg_Pre	Vtg_LLN	***Currently on dialysis**	
TLC_Ref	TLC_ULN	Creatinine level measured	
VC_Ref	VC_ULN	Last creatinine level	
FRCPL_Ref	FRCPL_ULN	Hemoglobin level measured	
ERV_Ref	RV_ULN	Last hemoglobin level	
RV_Ref	RV/TLC_ULN	COPD	
RV/TLC_Ref	Vtg_ULN	Interstitial fibrosis	
Vtg_Ref	DLCO_LLN	Lung cancer primary	
TLC_Pre %Ref	DLCO_ULN	Lung cancer invasion of adjacent structures	
VC_Pre %Ref		Reoperation	
FRCPL_Pre%Ref		Robotic technology-assisted	
ERV_Pre %Ref		ASA classification	
RV_Pre %Ref		Wedge	
Vtg_Pre %Ref		Segment	
DLCO_Pre		Lobectomy	
DL_Adj_Pre		Laterality	
DLCO/VA_Pre		Preoperative ejection fraction	
		History of Myocardial Infarction	
		Afib per EKG within the last year	

Figure S1. Sequential Feature Selector (SFS) curve for determination of optimal number of features to include in model. Marginal increases after 10 variables indicated that only the top 10 features should be included.

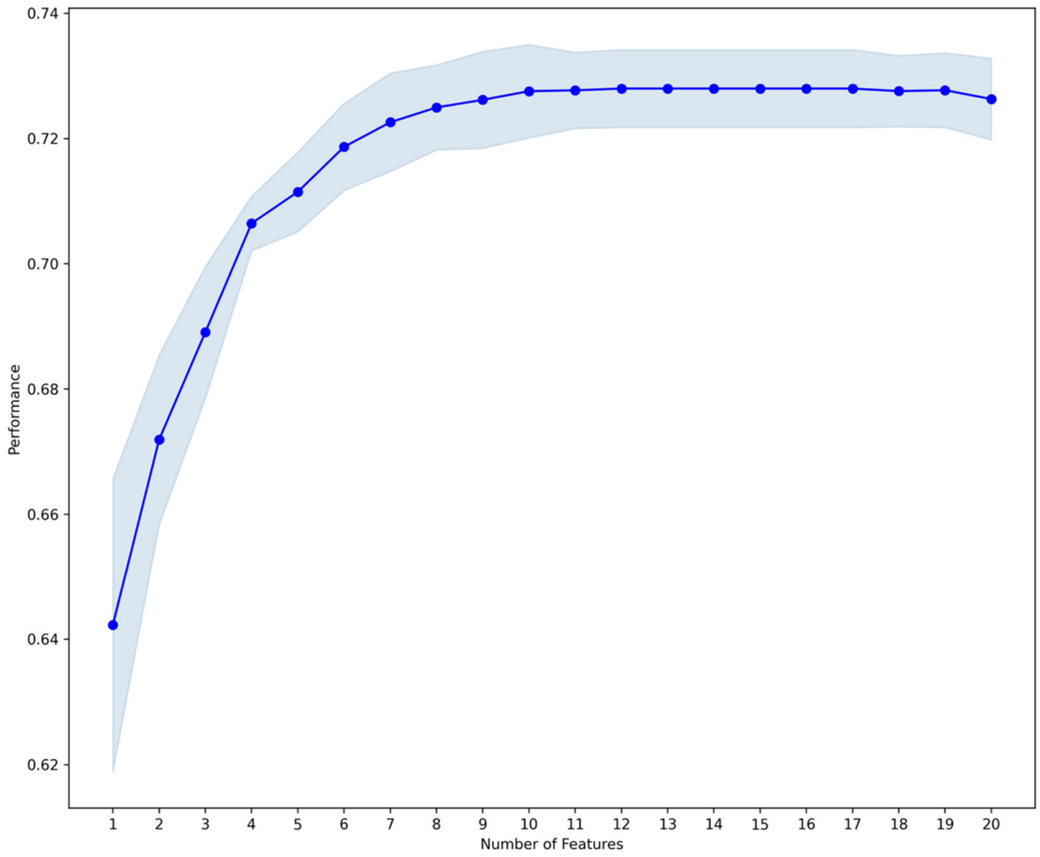

Figure S2. Artificial intelligence model reliability curves (A) Before calibration (B) After calibration

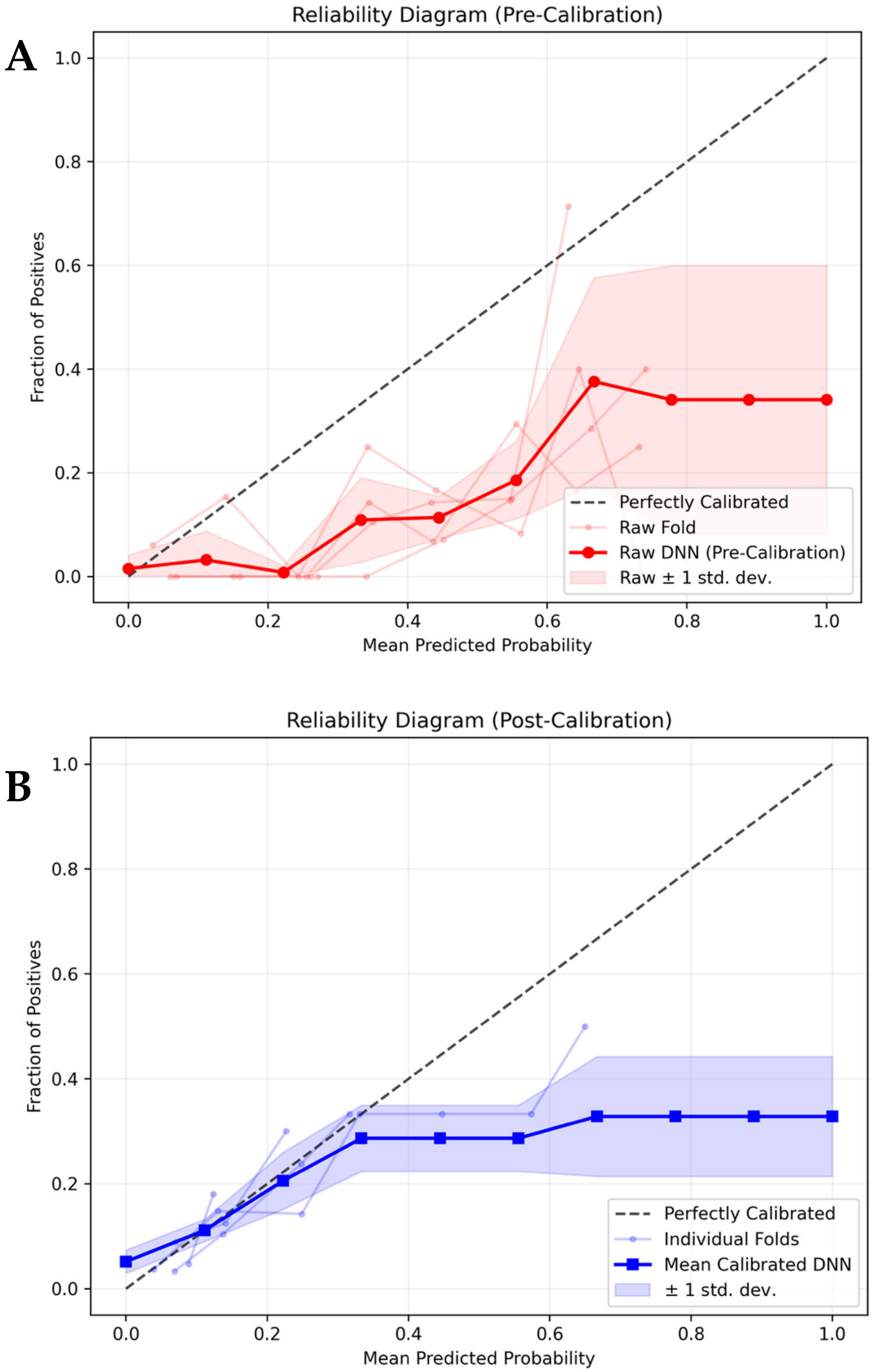

Figure S3. SHAP (Shapley Additive exPlanations) values for primary contributors to neural network model. Those not shown contributed zero or near-zero values, indicating that the L1 penalty effectively neutralized them.

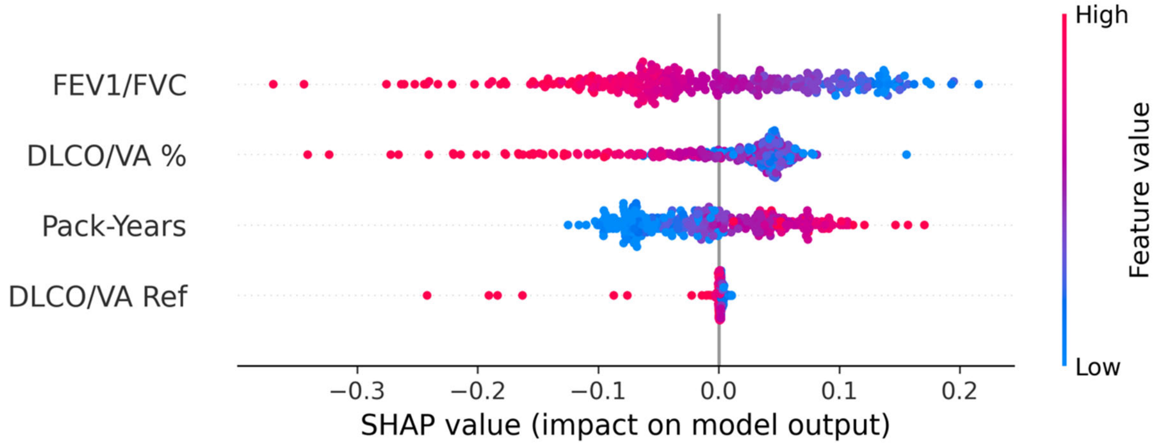

Supplement: Supplementary file 1 [file cancers-18-01484-s001.zip › cancers-4275600-supplementary.pdf]
